# Unilateral biportal endoscopic lumbar interbody fusion (ULIF) versus endoscopic transforaminal lumbar interbody fusion (Endo-TLIF) in the treatment of lumbar spinal stenosis along with intervertebral disc herniation: a retrospective analysis

**DOI:** 10.1186/s12891-024-07287-3

**Published:** 2024-02-29

**Authors:** Zuoran Fan, Xiaolin Wu, Zhu Guo, Nana Shen, Bohua Chen, Hongfei Xiang

**Affiliations:** 1 Department of Spine Surgery, The Affiliated Hospital of Qingdao University, Qingdao, Shandong 266000 China; 2 Department of Rehabilitation, The Affiliated Hospital of Qingdao University, Qingdao, Shandong 266000 China; 3 Department of Orthopedics, The Affiliated Hospital of Qingdao University, Qingdao, Shandong 266000 China

**Keywords:** Lumbar spinal stenosis, Unilateral biportal endoscopy technique, Endoscopic transforaminal lumbar interbody fusion, Health-related quality of life, Spinal canal volume, Rate of interbody fusion, Area of bone graft

## Abstract

**Objective:**

This study aims to compare the clinical effects and imaging data of patients who underwent endoscopic transforaminal lumbar interbody fusion (Endo-TLIF) with those who received unilateral biportal endoscopic lumbar interbody fusion (ULIF).

**Methods:**

A retrospective analysis was conducted on the clinical data of 69 patients presenting with typical intermittent claudication and signs and symptoms indicative of unilateral lower extremity nerve root compression, meeting inclusion criteria between April 2022 and June 2022. Among the cohort, 35 patients underwent ULIF group, while 34 patients underwent Endo-TLIF group. We compared perioperative parameters, including intraoperative blood loss, duration of hospital stay, and operation time between the two groups. Pre-operative and post-operative changes in the height and cross-sectional area of the target intervertebral space were also compared between the groups. Finally, we evaluated bone graft size and interbody fusion rates at 6 and 12 months post-surgery using the Brantigan scoring system.

**Results:**

The ULIF group had significantly shorter operative times compared to the Endo-TLIF group (*P* < 0.05). Conversely, the Endo-TLIF group exhibited significantly shorter hospital stays compared to the ULIF group (*P* < 0.05). However, there were no significant differences in intraoperative bleeding between the two groups (*P* > 0.05). Furthermore, both groups exhibited postoperative increases in vertebral canal volume compared to baseline (*P* < 0.05), with no significant difference in the change in the cross-sectional area of the target intervertebral space between the two surgical methods (*P* > 0.05). Interbody fusion rates were comparable between the two groups at both 6 and 12 months after surgery (*P* > 0.05). Lastly, the ULIF group had a significantly larger area of bone graft than the Endo-TLIF group (*P* < 0.05).

**Conclusion:**

In summary, the ULIF technique, as a novel spinal endoscopy approach, is a safer and more effective minimally invasive surgical method for addressing lumbar spinal stenosis and intervertebral disc herniation in patients. Both surgical methods have their own advantages and drawbacks. With the development of technology and related instruments, the limitations of both techniques can be mitigated for to a certain extent, and they can be applied by more doctors in diverse medical fields in the future.

## Background

With the acceleration of the aging process and improvements in living and working lifestyles among the Chinese population, the incidence of lumbar degenerative diseases has been steadily increasing. Among these conditions, the prevalence of lumbar spinal stenosis (LSS) in the elderly population is on the rise year by year. LSS encompasses bony spinal stenosis, spinal canal soft tissue hypertrophy, or a combination of both, leading to compression of the spinal cord, nerve roots, cauda equina nerves, and other structures. This compression can result in symptoms such as neurogenic claudication, radicular pain, and other manifestations of the syndrome. LSS is a frequently encountered degenerative condition of the lumbar spine that significantly impacts patients’ quality of life and motor function [1]. Currently, surgery is the most commonly applied method to alleviate symptoms of nerve root pain and intermittent claudication in patients with lumbar spinal stenosis [2]. To reduce surgical trauma and recovery time, an increasing number of spinal surgeries are now being performed using minimally invasive spinal endoscopy. Compared to conventional spinal surgery, spinal endoscopic surgery offers advantages such as reduced risk of bleeding, shorter hospital stays, smaller incisions, and minimal tissue damage. However, mastering and performing single-channel endoscopy can be challenging due to the limited intraoperative visual field of traditional endoscopy [3]. In addition, single-channel endoscopic inter-lumbar fusion is constrained by the size of the intervertebral fusion cage, as the fusion device must pass through a working cannula during surgery. In recent years, unilateral biportal endoscopy (UBE) spinal surgery technology has advanced in conjunction with the growth and refinement of minimally invasive surgical techniques. This approach not only allows for comprehensive decompression of the central spinal canal, bilateral nerve roots, and lateral recesses but also facilitates intervertebral bone graft fusion. Consequently, it has found increasing application in the clinical treatment of various spinal surgical conditions, yielding favorable outcomes [4].

While both endoscopic transforaminal lumbar interbody fusion procedures have been reported to yield favorable clinical results [5–7], studies assessing the extent of intervertebral space management and the efficacy of intervertebral fusion using these two methods are limited [8, 9]. Therefore, this study aims to compare the clinical and radiological outcomes of the these two minimally invasive decompression fusion procedures and explore their clinical effectiveness and postoperative imaging results.

## Materials and methods

### Patients

Patients diagnosed with spinal stenosis and concurrent intervertebral disc herniation who underwent minimally invasive intervertebral fusion in the Affiliated Hospital of Qingdao University from April 2022 to June 2022 were eligible for inclusion in this study. A portion of the patients included in the study were patients with discogenic spinal stenosis, while the other portion were patients with listhesis. All patients have lumbar spinal stenosis accompanied by intervertebral disc herniation, and their limping symptoms and leg pain can be alleviated solely through endoscopic decompression. However, given that patients also experience mechanical back pain related to stenosis symptoms, lumbar spine decompression surgery alone may not be effective, and the likelihood of postoperative symptom recurrence is high. Additionally, patients express a strong willingness to undergo lumbar fusion surgery. Therefore, all patients have surgical indications for undergoing lumbar fusion surgery. The average duration of follow up for two groups of patients ranged from preoperative to 1 year post-surgery, and average duration of follow up for two groups of patients was 12 months. The trial protocol received review and approval from the Medical Ethics Committee.

Inclusion Criteria: 1)Preoperative examination of the patients revealed that all patients had indications for fusion surgery, such as lumbar instability or facet hypertrophy; 2) Patients experiencing single-segment intervertebral disc herniation along with lumbar spinal stenosis or lateral recess stenosis as confirmed by imaging examinations; 3) Patients exhibiting typical intermittent claudication along with unilateral nerve root compression of the lower extremities, and who did not observe significant improvement in symptoms after a minimum of three months of regular conservative treatment, with a Visual Analog Scale (VAS) score for lumbar pain of ≥ 4.

Exclusion criteria: (1) Patients diagnosed with infectious diseases stemming from thoracolumbar lesions; (2) Patients who had previously undergone revision surgery at the same spinal level; (3) Patients with cardiopulmonary dysfunction rendering them ineligible for surgical intervention.

A total of 69 cases were included, comprising 33 males and 36 females, with ages ranging from 45 to 80 years. All the participants presented with typical radiating lower limb pain and intermittent claudication, with the ability to walk a distance of less than 100 m. Prior to inclusion, all patients underwent lumbar frontal and lateral radiography, lumbar hyperextension and hyperflexion lateral radiography, as well as lumbar CT and MRI examinations, revealing lumbar degeneration, lumbar disc herniation, and spinal stenosis. Specifically, 37 patients exhibited lumbar pathology at the L4/5 segment, while 32 patients were affected at the L5/S1 segment. The study received approval from the hospital ethics committee, and informed consent was obtained from patients and their families. Patients in both groups were operated on by the doctors from the same medical group with extensive experience in spinal surgeries and were divided into the ULIF and Endo-TLIF groups in accordance with their surgical modality. The participating doctors involved in the research are equally proficient in ULIF surgery and Endo-TLIF surgery, and have rich surgical experience in both types of surgery.

### ULIF

**Fig. 1:**
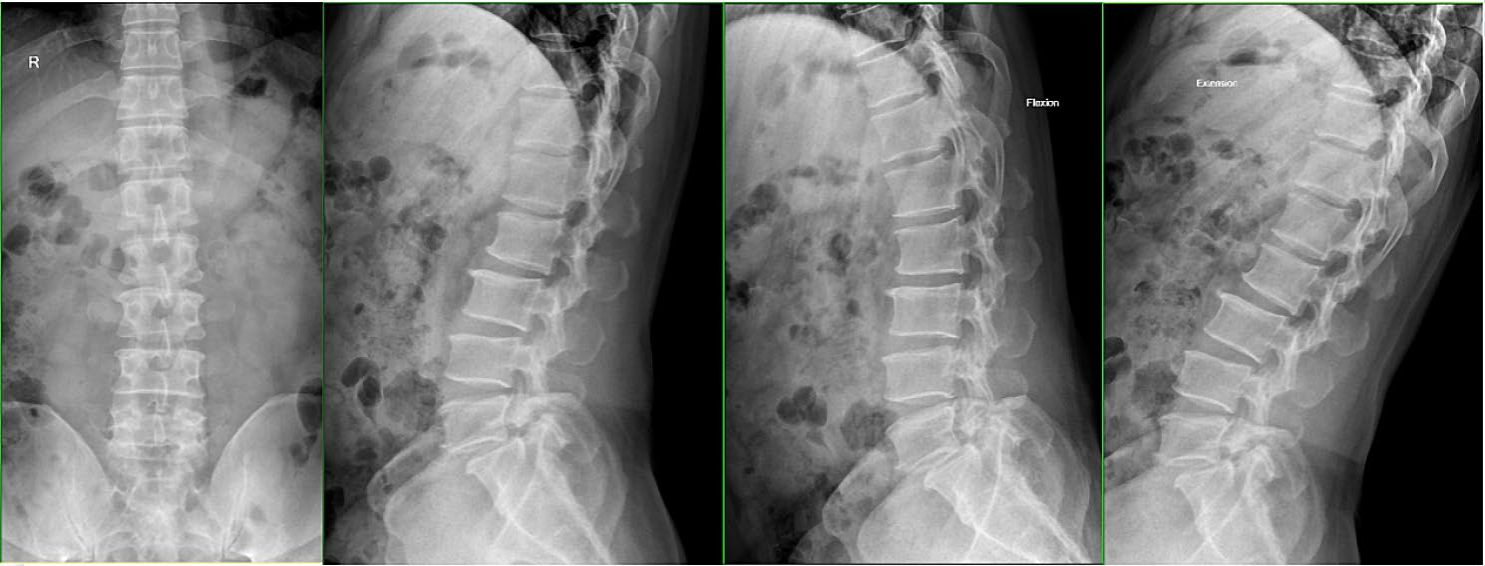
Pre-operative DR front-lateral and hyperflexion-hyperextension images of a patient in the ULIF group

**Fig. 2:**
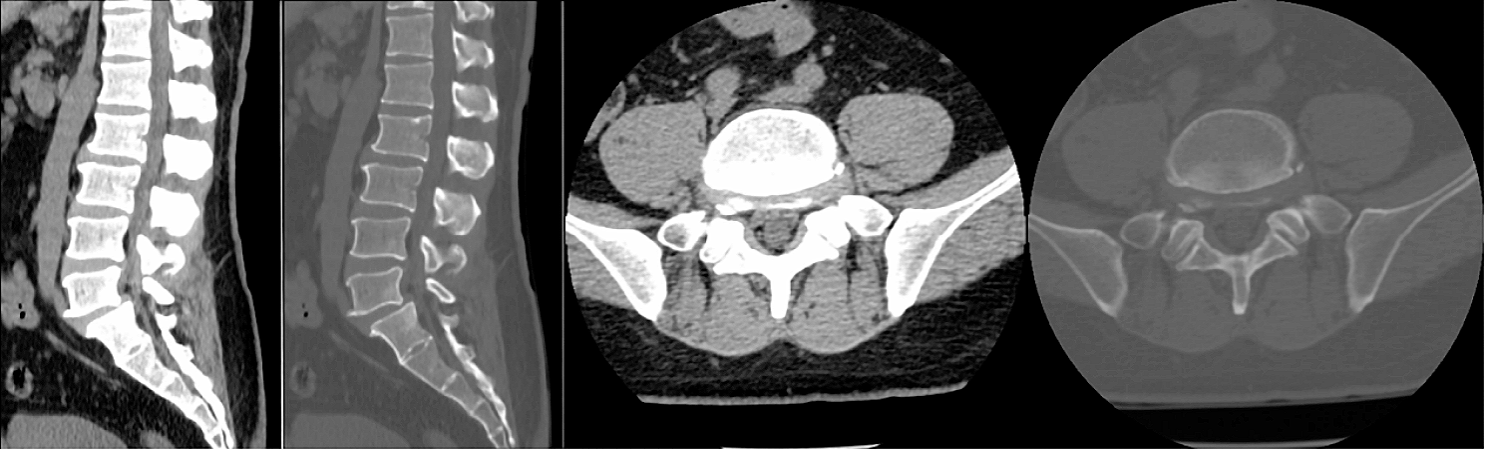
Pre-operative CT bone and soft tissue window of a patient in the ULIF group

**Fig. 3:**
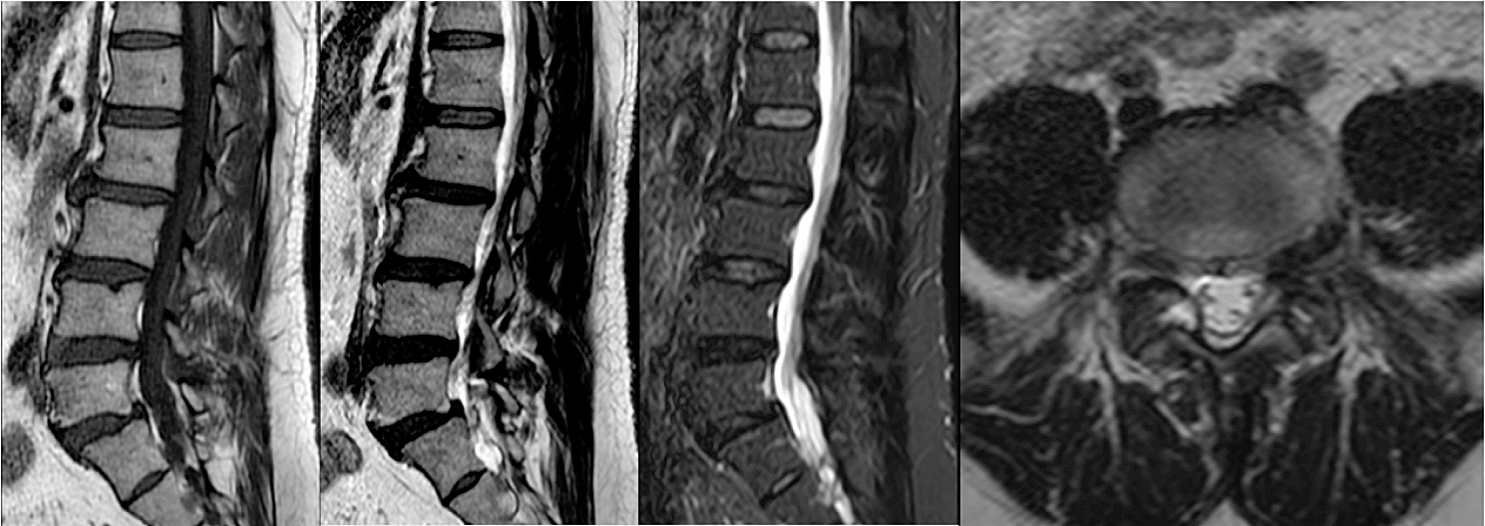
Pre-operative MR images of a patient in the ULIF group

**Fig. 4:**
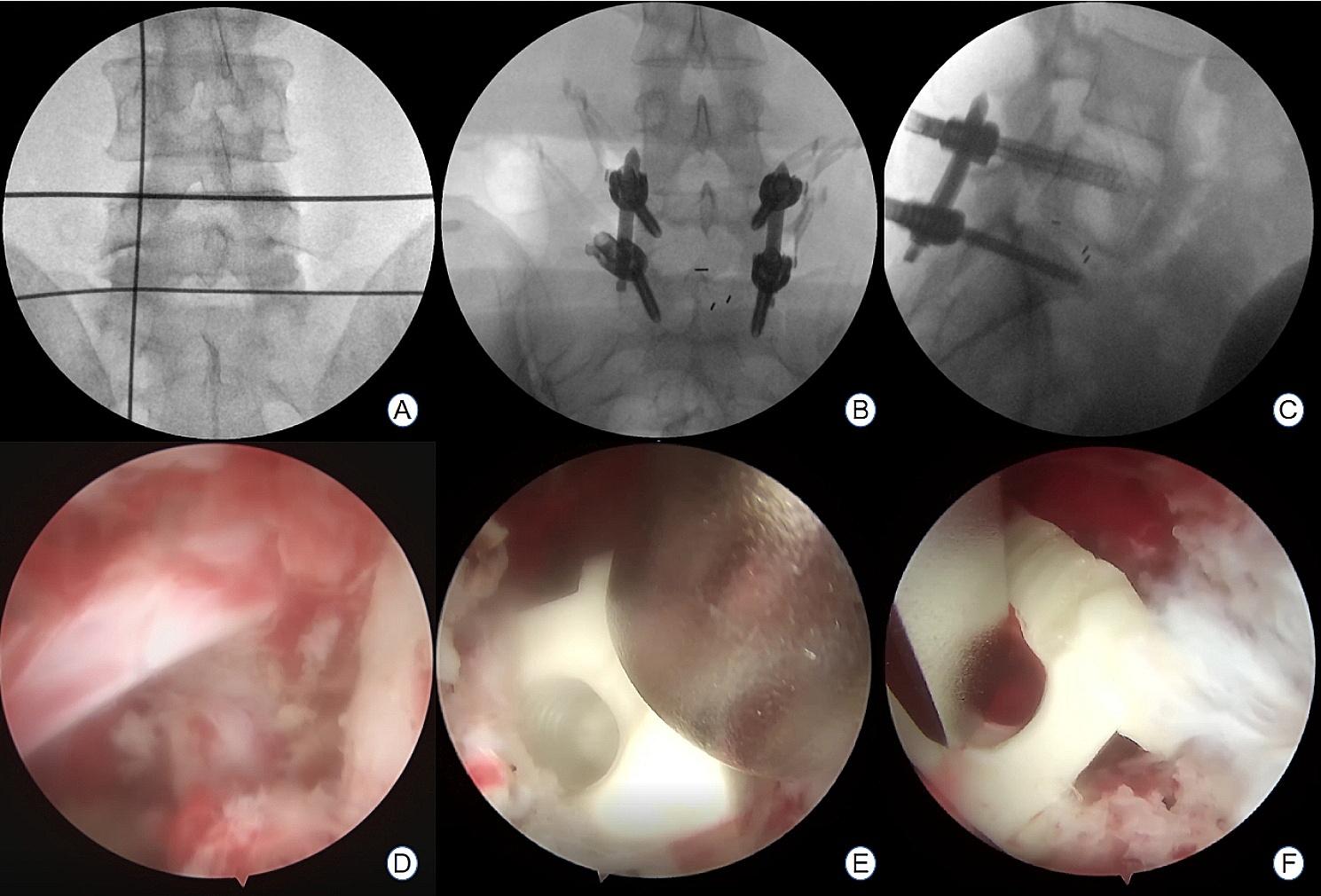
**A** Preoperative C-arm positioning in a patient at the L5/S1 segment; **B**, **C** C-arm fluoroscopic view of a patient with L5/S1 disc herniation after implantation of screws and nail rods; **D** Intraoperative exploration showing nerve root relaxation; **E**, **F** Cage implantation in the target vertebral space

**Fig. 5:**
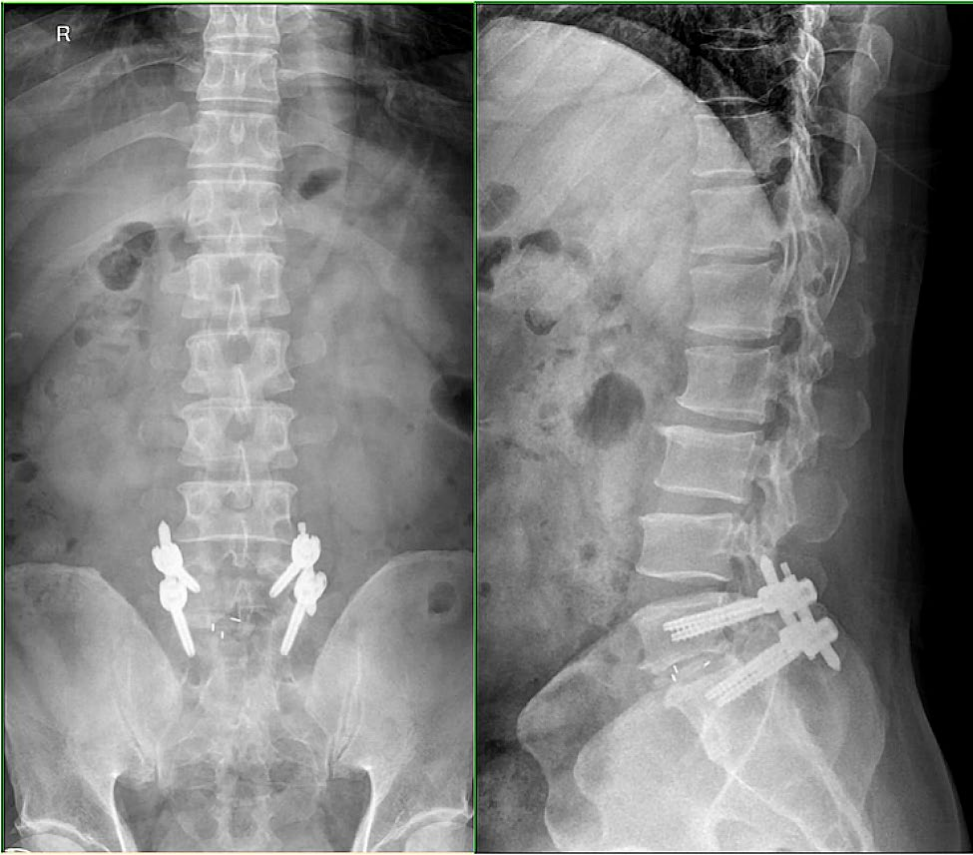
Postoperative DR frontal and lateral images of a patient in the ULIF group

**Fig. 6:**
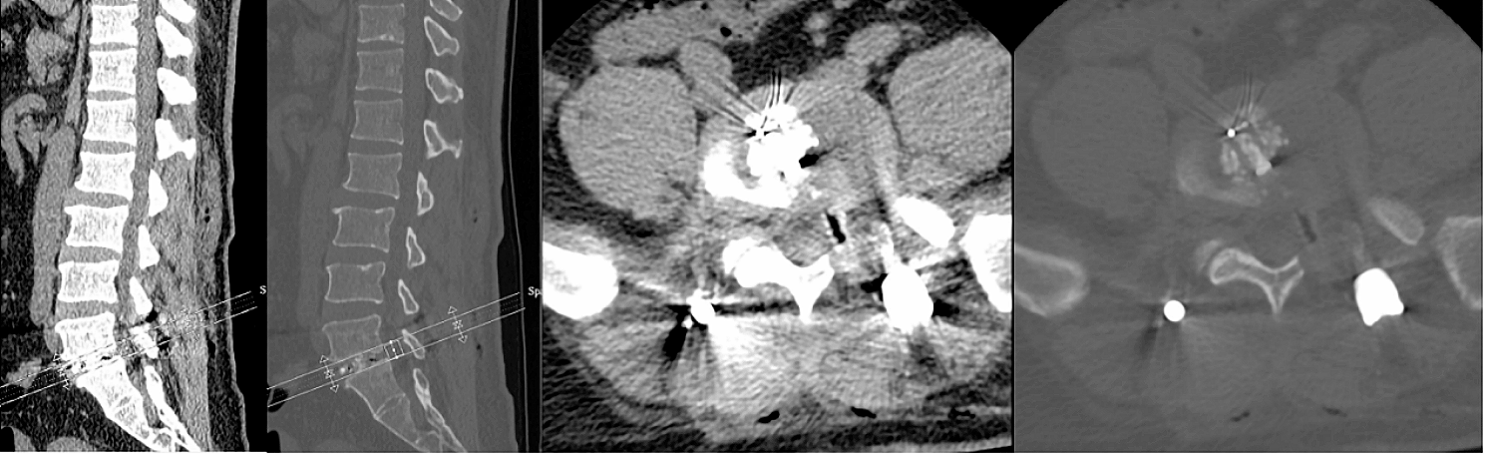
Postoperative CT bone and soft tissue window of a patient in the ULIF group

**Fig. 7:**
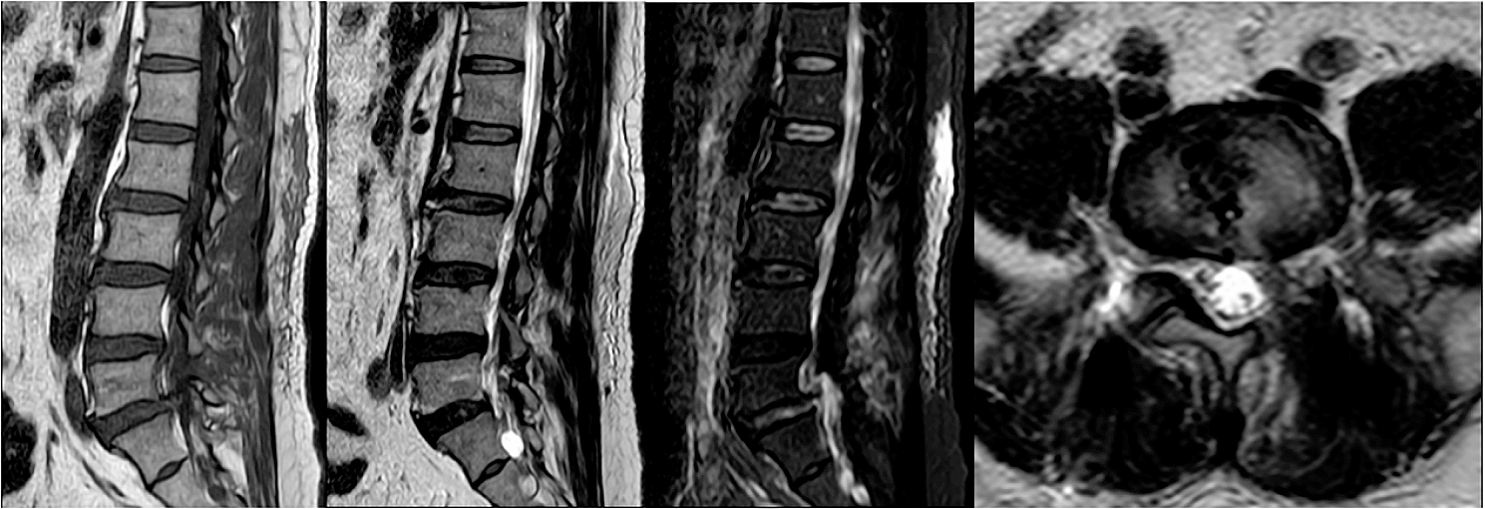
Postoperative MR image of a patient in the ULIF group

A total of 35 patients were allocated to the ULIF group, and the preoperative imaging results for a patient are presented in Figs. 1, 2 and 3. Following successful general anesthesia, the patient was positioned in the prone orientation on the operating table with submental padding. Based on the more severe side of the patient’s symptoms (in cases of asymmetrical symptoms), C-arm X-ray fluoroscopy was employed to locate the ipsilateral intervertebral space of the target segment, with the puncture point situated approximately 0.5 cm adjacent to the spinous process (Fig. 4A).Standard skin disinfection procedures were performed, and a sterile cavity towel was placed. After successful electromagnetic navigation alignment, a guide wire was bilaterally inserted into the target segment of the vertebral arch. Subsequently, a puncture needle was used, guided by fluoroscopy, to establish bilateral access to the UBE (Unilateral Biportal Endoscopy) system, connecting it to the light source and camera. The light source was then activated, and color balance adjustments were made to achieve optimal visualization. Following this, the intervertebral foramoscope was introduced into the working cannula, and water flow was adjusted. A fluid pump was used and at 30 mmHg. Bone on the medial aspect of the ipsilateral inferior articular eminence of the superior vertebral body and bone on the medial aspect of the superior articular eminence of the inferior vertebral body were partially excised within the target intervertebral space. Only a part of the superior articular process was removed. Following unilateral facet joint resection, place the UBE retractor through the working portal to pull the nerve root toward the midline, and place the nucleus pulposus forceps along the UBE retractor through the working portal to remove the protruding nucleus pulposus tissue. Dissected the adhesion tissue with a nerve dissector, and use the radiofrequency electrocautery locally to achieve hemostasis until the nerve roots are freed with good mobility, preventing compression of the nerve root and dura mater upon exploration. The herniated nucleus pulposus was removed through the working channel while carefully probing the dural sac (Fig. 4B). Moreover, the target intervertebral space was treated, and an autologous granulated bone was implanted for compaction along with a carbon fiber cage of appropriate size (Fig. 4C), with adjustments made to its positioning and orientation. Radiofrequency bipolar electrodes were introduced through the working channel to control bleeding and ablate the nucleus pulposus. Subsequently, four pedicle screws were inserted in the bilateral pedicle under C-arm fluoroscopy (Fig. 4D–F). Following this, the working channel was withdrawn, a drainage tube was placed, and the surgical wound was sutured. Finally, the skin was disinfected, and the a sterile dressing was applied. The patient reported no discomfort, and no abnormalities were observed in the sensory movements of both lower extremities post-operation. The patient was then transferred to the ward on a flatbed, with stable vital signs. Postoperative imaging data for a patient are depicted in Figs. 5, 6 and 7.

### Endo-TLIF

**Fig. 8:**
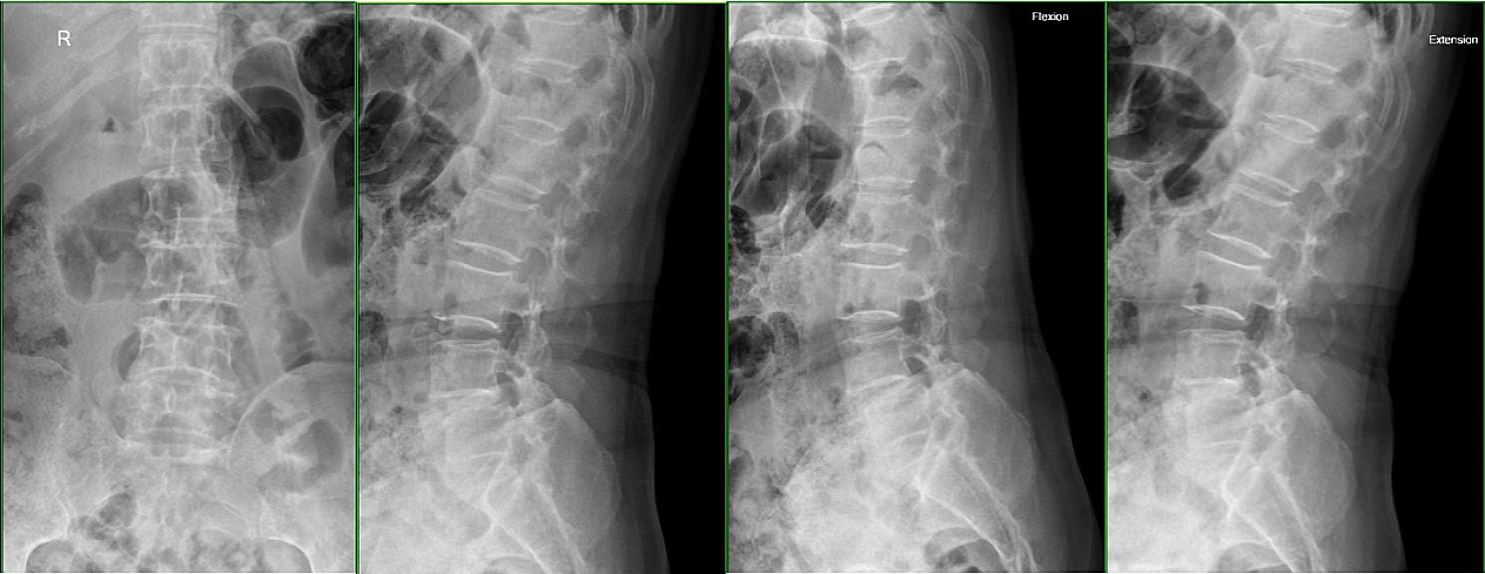
Pre-operative DR front-lateral and hyperflexion-hyperextension images of a patient in the Endo-TLIF group

**Fig. 9:**
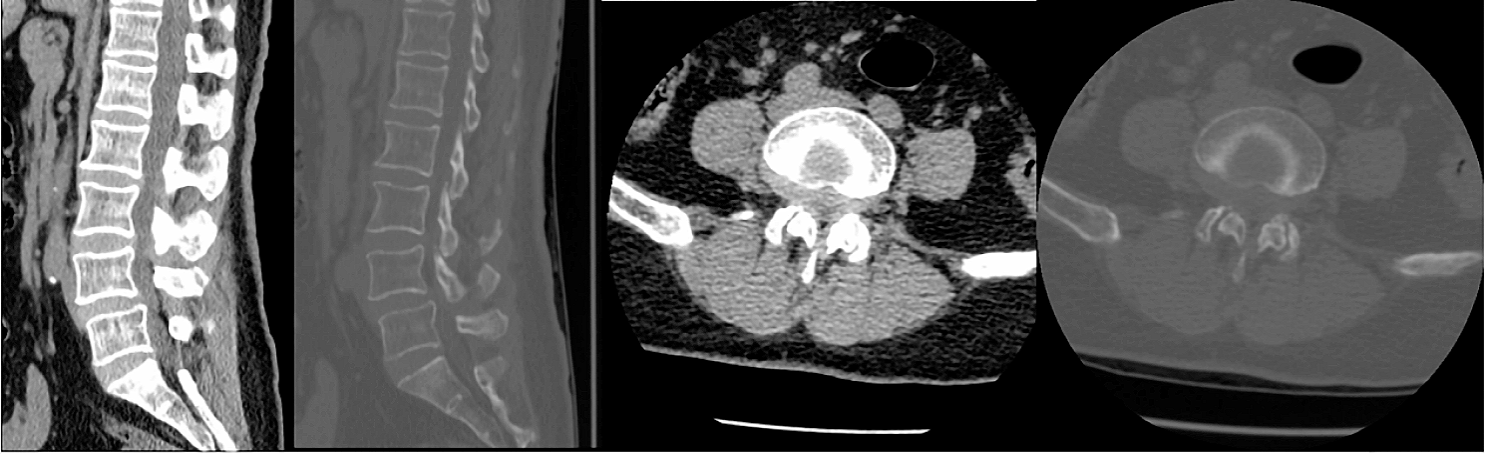
Pre-operative CT bone and soft tissue window of a patient in the Endo-TLIF group

**Fig. 10:**
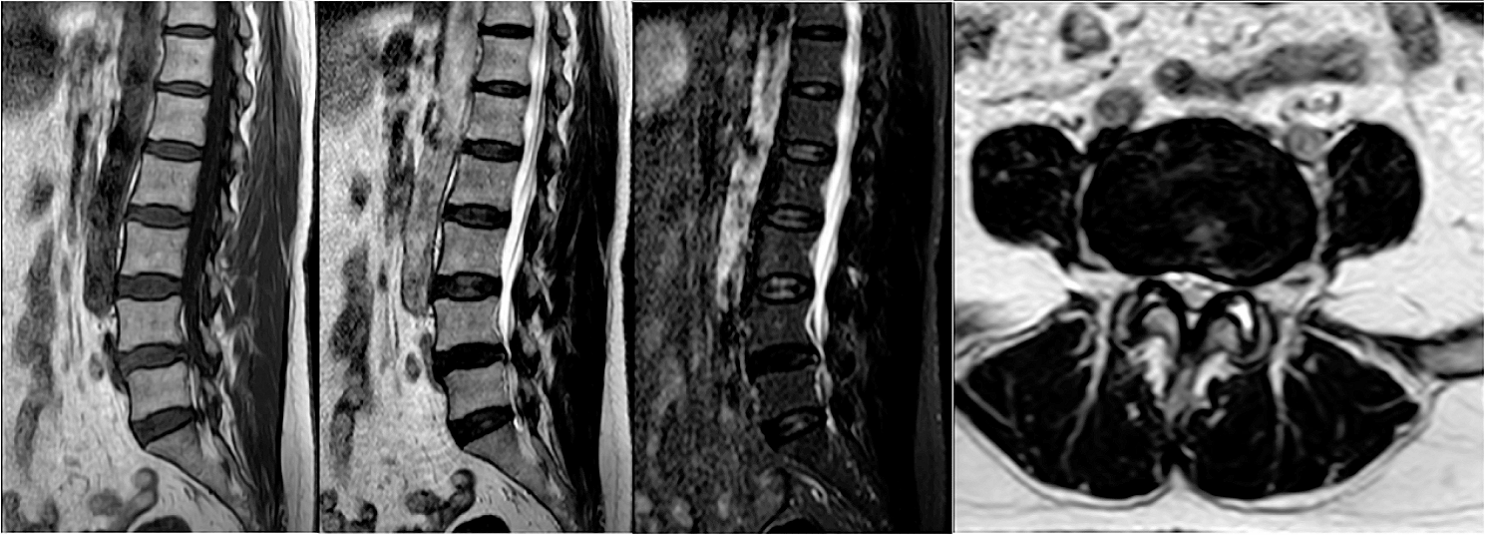
Pre-operative MR images of a patient in the Endo-TLIF group

**Fig. 11:**
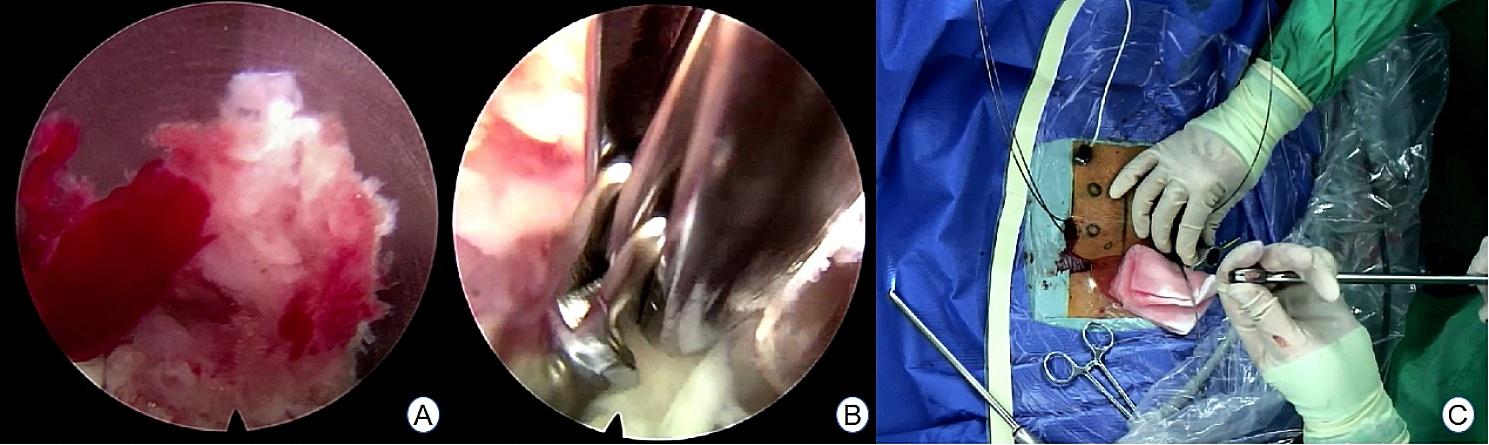
**A** Endoscopic trepanation during the Endo-TLIF procedure; **B** Treatment of the intervertebral space during the ENDO-TLIF procedure; **C** Cage implantation during the Endo-TLIF procedure

**Fig. 12:**
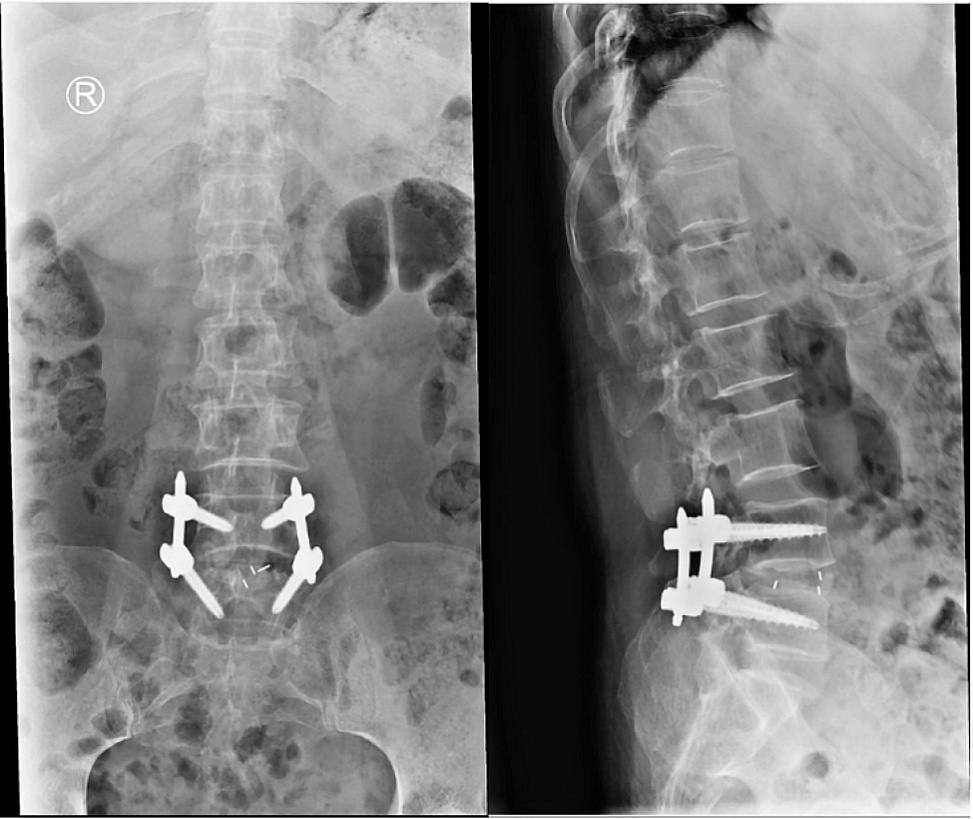
Postoperative DR frontal and lateral images of a patient in the Endo-TLIF group

**Fig. 13:**
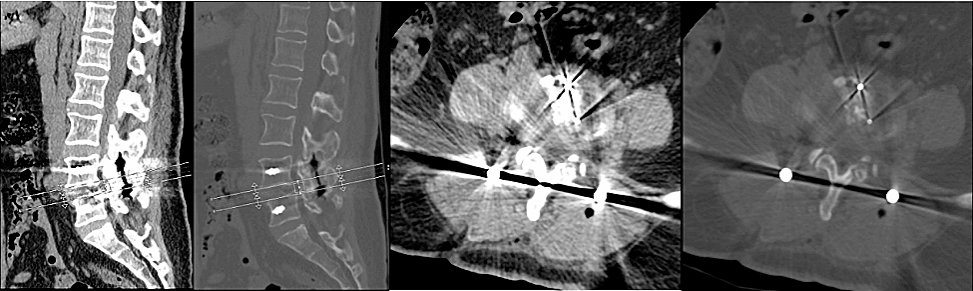
Postoperative CT bone and soft tissue window of a patient in the Endo-TLIF group

**Fig. 14:**
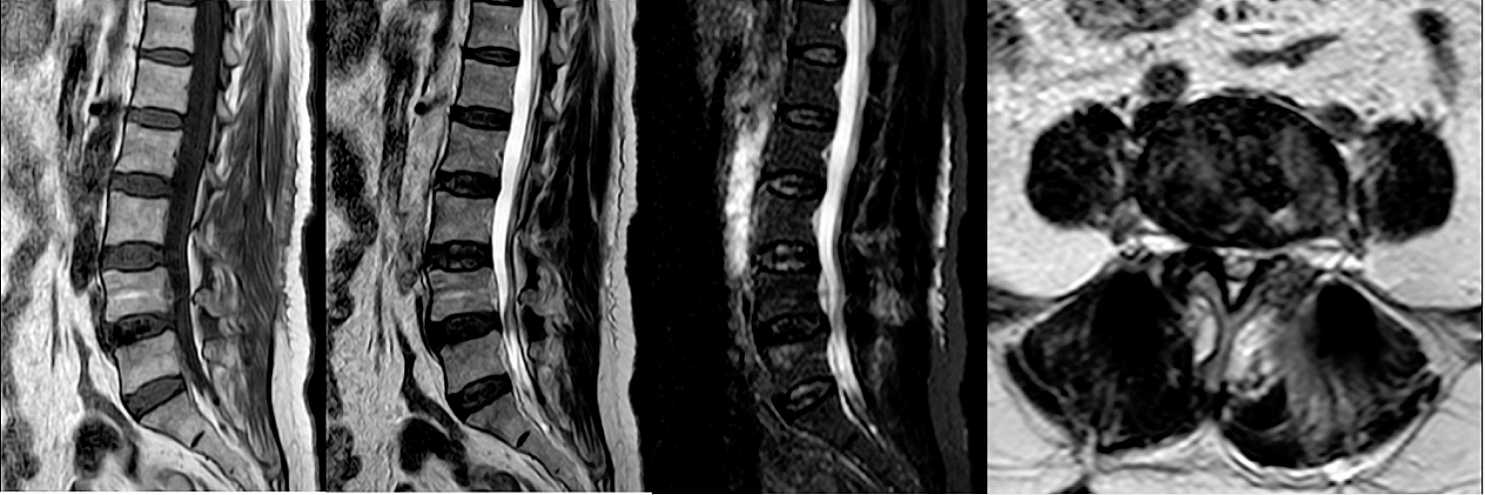
Postoperative MR image of a patient in the Endo-TLIF group

The Endo-TLIF group comprised 34 patients, and postoperative imaging data for one patient are presented in Figs. 8, 9 and 10. Following successful general anesthesia, the patient was transported to the operating room and positioned in the prone posture with adequate chest and skeletal padding. Similar to the ULIF group, four guide wires were inserted into the bilateral pedicles under fluoroscopic guidance targeting the specific segment, with the assistance of electromagnetic navigation. The target vertebral space was identified on the ipsilatera side of the intervertebral space using electromagnetic navigation, and the puncture site was chosen at the level of the flat intervertebral space. The accuracy of the puncture was verified through frontal and lateral fluoroscopy, confirming placement at the ipsilatera superior articular eminence of the inferior vertebral body. Subsequently, a guide wire was inserted, the puncture needle was removed, and an incision of approximately 8 mm was made at the entry point. A guide rod was subsequently introduced along the guide wire, and finally, a working trocar was placed. C-arm fluoroscopy confirmed the proper positioning of the working trocar, after which the guide wire and rod were withdrawn. Following this, the intervertebral foramoscope was connected and flushed with saline. The light source was activated, and color balance adjustments were made to achieve optimal visualization. The intervertebral foramoscope was inserted into the working trocar, and the water flow was adjusted. Muscles, fatty tissues, and other soft tissues in the field of view were retracted, and bleeding was cauterized by radiofrequency. Preprocess the surface bone using a ring saw or large nucleus pulposus forceps until a clear joint surface is exposed. The apex of the upper articular process, along with some lower articular processes, serves as the site for the first use of a circular saw to remove a portion of the articular process, so as to expose the structure of the ligamentum flavum directly (Fig. 11A). The adjacent ligamentum flavum was also excised, allowing for decompression of the lateral recess and intervertebral foramen. The target intervertebral space was then identified, and posterior herniation of the disc was managed. Subsequently, meticulous removal of the nucleus pulposus was performed using grasping forceps (Fig. 11B). The upper and lower endplates were scraped off with a working trocar spinotomy, and an occluded fragmented bone and a portable cage were implanted, propped, and fixed at the appropriate height in the anterior column through the open intervertebral foramen and trans kambin route(Fig. 11C). Adequate inflation of the dural sac was ensured, and any remnants of the nucleus pulposus within the spinal canal were removed. The nail tract was dilated using wiretapping along the guide wire, and four pedicle screws were inserted under fluoroscopy. The bilateral pedicles were connected with longitudinal rods and locked by screw caps of appropriate length. Finally, the wound was sutured, and a sterile dressing was applied.No indwelling drainage tube after surgery.The procedure proceeded smoothly, with satisfactory anesthesia outcomes. Following the operation, the patient was transferred to the ward. Postoperative imaging results for a patient are depicted in Figs. 12, 13 and 14.

### Postoperative management methods

Drainage tube removal time: In the ULIF group, the timing for the removal of the drainage tube should be determined on a case-by-case basis, typically occurring 2–3 days after the surgical procedure and the postoperative drainage volume of this group of patients is 30-80 ml, with an average of 50 ml.The decision will be made by the medical team based on postoperative drainage levels and the patient’s progress in rehabilitation.

Postoperative rehabilitation: Both groups of patients adopted the same rehabilitation protocol after surgery and both study groups exhibited similar postoperative rehabilitation outcomes, with improvements noted in VAS scores for low back pain and leg pain, as well as in the Oswestry Dysfunction Index. Postoperative rehabilitation is a gradual process wherein patients are advised to incrementally increase their physical activity and engage in rehabilitation exercises under the guidance of their healthcare providers.

### Parameters measurement

Patient information and treatment efficacy were systematically followed up and documented. The relevant measurements in this study were taken by three blinded assessors, and the fourth person collected the measurement results of the same indicator from three individuals on the same patient, and took the average value, Overall treatment efficacy was evaluated by recording and comparing the VAS scores and ODI index for low back pain and leg pain before surgery and at 1 week, 6 months, and 12 months postoperatively. (2) Both patient groups underwent lumbar spine MRI plain scans one day before the surgical procedure and one week after surgery. The level of middle disc was selected as the measurement plane. Image J software was utilized to measure the volume of the spinal canal in the same plane before and after surgery. Changes in measurements before and after surgery were calculated to evaluate the decompression effect. (3) One week post-surgery, both patient groups underwent lumbar CT three-dimensional imaging examinations. Using Image J software, the irregular bone graft area of each plane within the target intervertebral space was measured, and the maximum bone graft area was selected for comparison. (4) Additionally, both groups underwent lumbar CT three-dimensional imaging examinations one day before surgery and one week post-surgery. The height of the target segment’s intervertebral space was measured, and changes before and after surgery were calculated for comparison and statistical analysis. (5) Intervertebral fusion in both groups of patients was assessed post-surgery using the modified Brantigan scoring system. The scoring system is as follows: 4 points for complete fusion with continuous callus; 3 points for good intervertebral fusion with faint transparent lines; 2 points for continuous callus present in 50% of the intervertebral space, with numerous transparent lines in the intervertebral implanted bones; 1 point for no continuous callus in most of the intervertebral implanted bones, but an increase in bone volume compared to postoperative intervertebral implanted bone; and 0 points for intervertebral implanted bone resorption, reduced intervertebral space height, and lack of vertebral body fusion. The fusion criteria were defined as a modified Brantigan score ≥ 3 points. (6) Calculation of intraoperative blood loss: In this study, two groups of intraoperative blood loss = the difference in weight of gauze before and after surgery + the amount of fluid in the drainage tube + total intraoperative fluid outflow after continuous flushing- Intraoperative flushing saline volume. (7) Physical examinations were conducted on both groups of patients before and one week after surgery. The included items in the physical examination were (A) straight leg elevation test, (B) strengthening test, (C) lower limb muscle strength, (D) spinal buckle pain, (E) knee reflex, (F) ankle reflex, (G) spinal range of motion, and (H) sensory impairment.

### Statistical analysis

Statistical analysis was conducted using SPSS 27.0. The normality of the different variables was checked by Shapiro-Wilk and Kolmogorov-Smirnova tests, Continuous variables with a normal distribution were presented as means ± standard deviation ($\bar{x}$ ± s) and intergroup comparisons were carried out using the t- test.Non-normally distributed variables of continuity are represented by the median (quartile),and intergroup comparisons were carried out using the Mann-Whitney U test.For categorical variables we use N (%) to represent,and intergroup comparisons were carried out using the χ^2^ test. Repeated measures analysis of variance was utilized to compare two groups across multiple time points. In cases where the sphericity assumption was not met, the Greenhouse-Geisser method was applied for correction. The Bonferroni method was used for comparison between different time points in the same group, and the multiple factor analysis of variance was used for comparison between different groups at the same time point. The significance level was set at α = 0.05.

## Results

The age distribution of patients in both groups exhibited a normal distribution, and baseline characteristics were comparable between the two groups (*P* > 0.05), as summarized in Table 1. Preoperative imaging examinations of two groups of patients showed that both groups belonged to patients with severe lumbar spinal stenosis. During the perioperative period, patients were carefully examined and followed up. The operative time, intraoperative blood loss, and length of hospital stay for each group of patients are summarized in Table 2. It is important to note that the data from both groups exhibited a normal distribution. Consequently,the independent sample t-test was applied for comparative analysis. The results revealed a significantly shorter operative time in the ULIF group when compared to the Endo-TLIF group (*P* < 0.05). Moreover, There is no significant difference in intraoperative bleeding between the two groups of patients (*P*>0.05). Conversely, the duration of hospital stay was significantly longer for patients in the ULIF group in comparison to those in the Endo-TLIF group (*P* < 0.05) (Table 2).The results of the physical examinations are shown in Table 3. Upon comparing the preoperative and postoperative examination results, significant recovery was observed in the straight leg elevation test, strengthening test, muscle strength, and spinal buckle pain of the two groups of patients (*P* < 0.05); However, there was no notable improvement in knee reflex, ankle reflex, spinal range of motion, and sensory impairment between the two groups of patients (*P* > 0.05). Additionally, there was no statistically significant difference between the two groups of patients (*P* > 0.05). In this study, two cases of postoperative cerebrospinal fluid leakage were reported in the Endo-TLIF group patients. These patients were treated with 1 needle of surgical incision pressure suture and enhanced dressing change. Symptoms improved 5 days after surgery; In the ULIF group, one patient experienced postoperative cerebrospinal fluid leakage, and one needle of pressure suture was given to the drainage tube opening. The patient’s symptoms improved 3 days after surgery; Both groups of patients showed no signs of nerve root injury or epidural hematoma, and there was no infection after surgery. No statistically significant difference was identified between the two groups of patients (*P* > 0.05).

**Table 1:** Comparison of baseline data between groups

Baseline data	ULIF(*n* = 35)	Endo-TLIF(*n* = 34)	Statistic	P-value
Gender (male, cases (%))	17 (48.57)	16 (47.06)	*χ*^2^ < 0.001	> 0.999
Age ($\bar{x}$ ± s, years)	58.80 ± 11.21	60.00 ± 10.71	*t*=-0.455	0.651
Course of disease ($\bar{x}$ ± s, months)	11.16 ± 2.20	11.96 ± 2.21	*t*=-1.499	0.139
Lesion segment (L4/5, cases (%))	19 (54.29)	18 (52.94)	*χ*^2^ < 0.001	> 0.999
VAS preoperative score for leg pain M (Q1,Q3)	7.00(6.00,7.00)	6.00(5.15,7.00)	Z=-0.617	0.537
VAS preoperative score for low back pain M (Q1,Q3)	6.50(5.75,7.50)	6.00(5.00,7.00)	Z=-0.590	0.555
VAS score for back pain : VAS score for leg pain M (Q1,Q3)	1.00(0.93,1.12)	1.00(0.81,1.16)	Z = 0.000	0.619
Preoperative ODI index	65.03 ± 7.99	59.21 ± 6.46	*t* = 3.334	0.001
Preoperative spinal canal area ($\bar{x}$ ± s,mm^2^)	0.66 ± 0.22	0.67 ± 0.23	t = 0.014	0.7927
Preoperative intervertebral space height M (Q1,Q3)(mm)	8.26(7.20,9.09)	8.26(6.67,10.21)	Z=-0.234	0.815

**Table 2:** Comparison of outcome indicators between groups

Outcome indicators	ULIF(*n* = 35)	Endo-TLIF(*n* = 34)	Effect value (95% CI)	P-value
Surgical time ($\bar{x}$ ± s, min)	112.78 ± 19.29	174.58 ± 18.41	61.80 (52.90, 70.70)	< 0.001
Intraoperative bleeding volume ($\bar{x}$ ± s, mL)	97.71 ± 11.15	101.73 ± 9.74	4.02 (-0.92, 8.97)	0.116
Hospitalization time ($\bar{x}$ ± s, d)	6.81 ± 2.21	5.13 ± 2.03	-1.68 (-2.69, -0.68)	0.002
Intervertebral space bone graft area ($\bar{x}$ ± s, mm^2^)	3.63 ± 0.44	2.20 ± 0.62	-1.43 (-1.68, -1.18)	< 0.001
Intervertebral fusion 6 months after surgery (yes,cases (%))	31 (88.57)	29 (85.29)	0.75 (0.18, 3.06)	0.705
Intervertebral fusion at 12 months after surgery (yes/cases (%))	33 (94.29)	32 (94.12)	0.97 (0.13, 7.31)	0.978
Postoperative intervertebral height ($\bar{x}$ ± s, mm)	10.49 ± 1.30	10.87 ± 1.78	0.38 (-0.36, 1.11)	0.316
Postoperative spinal canal area ($\bar{x}$ ± s, cm^2^)	1.67 ± 0.47	1.71 ± 0.47	0.037(-0.184, 0.259)	0.7415
Change in spinal canal area ($\bar{x}$ ± s, cm^2^)	1.02 ± 0.47	1.04 ± 0.49	0.023(-0.205, 0.252)	0.8417
Change in intervertebral space height ($\bar{x}$ ± s, mm)	2.29 ± 1.42	2.38 ± 1.60	0.09 (-0.62, 0.80)	0.808
VAS leg pain score 1 week after surgery M (Q1,Q3)	3.00(2.00,3.00)	2.00(2.00,3.00)	-0.14 (-0.50, 0.21)	0.439
VAS leg pain score 3 months after surgery M (Q1,Q3)	2.00(1.00,2.00)	1.00(1.00,2.00)	-0.26 (-0.57, 0.05)	0.087
VAS leg pain score 12 months after surgery M (Q1,Q3)	1.00(1.00,2.00)	1.00(1.00,2.00)	-0.05 (-0.37, 0.28)	0.760
VAS low back pain score 1 week after surgery M (Q1,Q3)	2.00(2.00,3.00)	2.00(2.00,3.00)	0.11 (-0.22, 0.44)	0.746
VAS low back pain score 3 months after surgery M (Q1,Q3)	2.00(1.00,2.00)	2.00(1.00,2.00)	0.09 (-0.27, 0.44)	0.499
VAS low back pain score 12 months after surgery M (Q1,Q3)	1.00(1.00,2.00)	1.00(1.00,2.00)	0.05 (-0.27, 0.37)	0.899
VAS score for back pain : VAS score for leg pain 1 week after surgery M (Q1,Q3)	0.86(0.67,1.00)	1.00(0.67,1.50)	0.189(-0.054, 0.432)	0.283
VAS score for back pain : VAS score for leg pain 3 months after surgery M (Q1,Q3)	1.00(0.50,1.00)	1.00(0.50,2.00)	0.182(-0.134, 0.499)	0.190
VAS score for back pain : VAS score for leg pain 12 months after surgery M (Q1,Q3)	1.00(0.50,1.00)	1.00(0.50,1.00)	-0.028(-0.312, 0.257)	0.722
ODI index 1 week after surgery ($\bar{x}$ ± s)	18.17 ± 4.35	19.09 ± 3.62	0.97(-2.84, 1.01)	0.345
ODI index 6 months after surgery ($\bar{x}$ ± s)	8.14 ± 2.68	8.06± 2.70	0.65 (-1.21, 1.38)	0.897
ODI index 12 months after surgery ($\bar{x}$ ± s)	7.17 ± 3.29	7.15 ± 2.80	0.74(-1.44, 1.49)	0.974

**Table 3:** Comparison of physical examination between groups

Abnormal physical examination results	ULIF(*n* = 35)		Endo-TLIF(*n* = 34)	
	Preoperative results	Preoperative results	Preoperative results	Preoperative results
Straight leg elevation test (cases (%))	20(57.14)	3(8.57)	18(52.94)	2(5.88)
Strengthening test (cases (%))	18(51.43)	2(5.71)	17(50)	2(5.88)
Lower limb muscle strength (cases (%))	28(80)	12(34.29)	26(76.47)	10(29.41)
Spinal buckle pain (cases (%))	25(71.43)	5(14.29)	24(70.59)	3(8.82)
Knee reflex (cases (%))	21(60)	20(57.14)	20(58.82)	20(58.82)
Ankle reflex (cases (%))	18(51.43)	18(51.43)	16(47.06)	15(44.12)
Spinal range of motion (cases (%))	23(65.71)	19(54.29)	22(64.71)	17(0.5)
sensory impairment (cases (%))	32(91.43)	31(88.57)	32(94.12)	30(88.24)

**Fig. 15:**
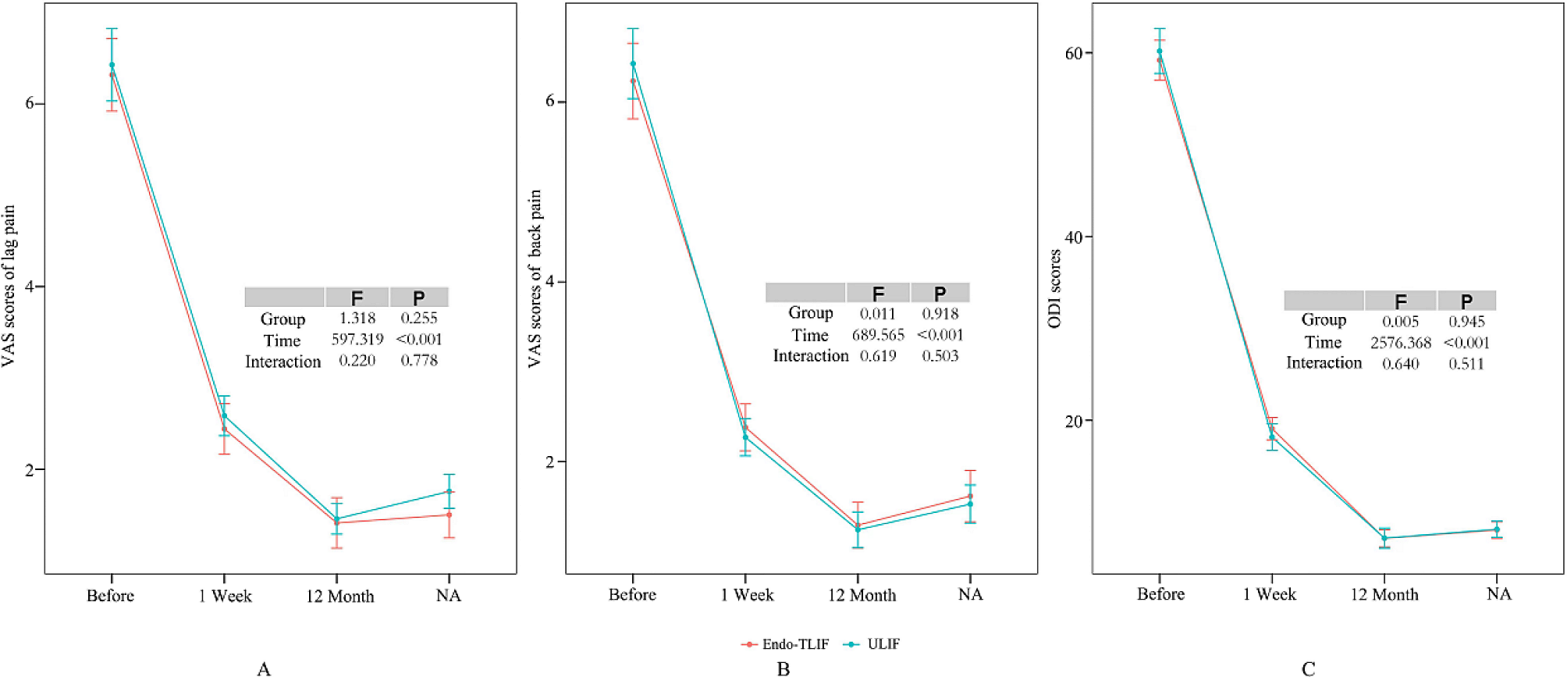
Comparison of therapeutic effects between two groups of patients **A** Changes in VAS scores for leg pain between the two groups of patients; **B** Changes in VAS scores for low back pain in two groups of patients; **C** Changes in ODI index between two groups of patients

**Fig. 16:**
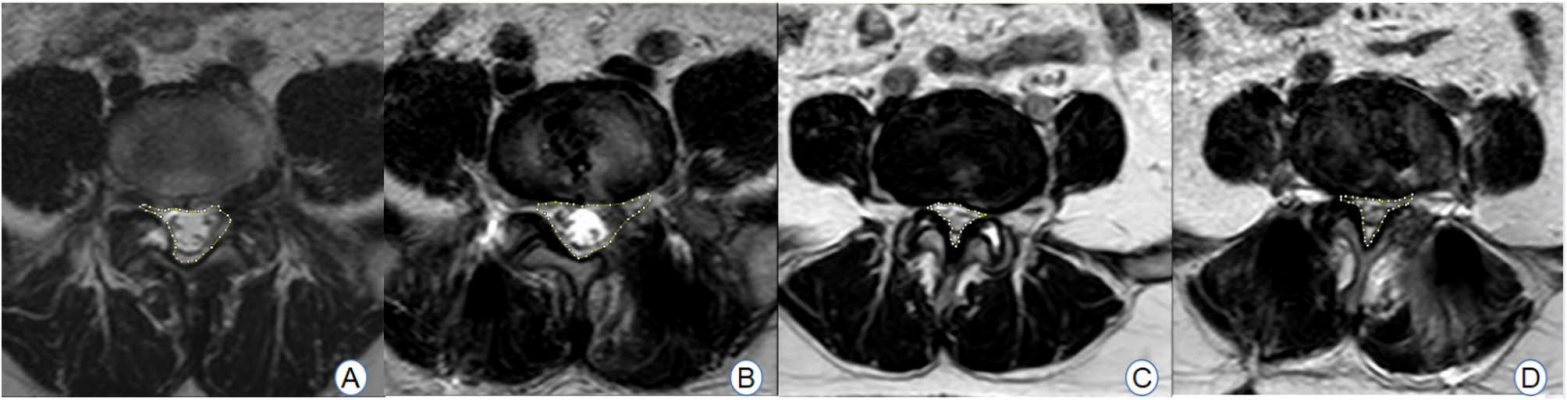
**A**, **B** Area of spinal canal measured by Image J before and after surgery in a patient of the ULIF group; **C**, **D** Area of spinal canal measured by Image J before and after surgery in a patient of the Endo-TLIF group

**Fig. 17:**
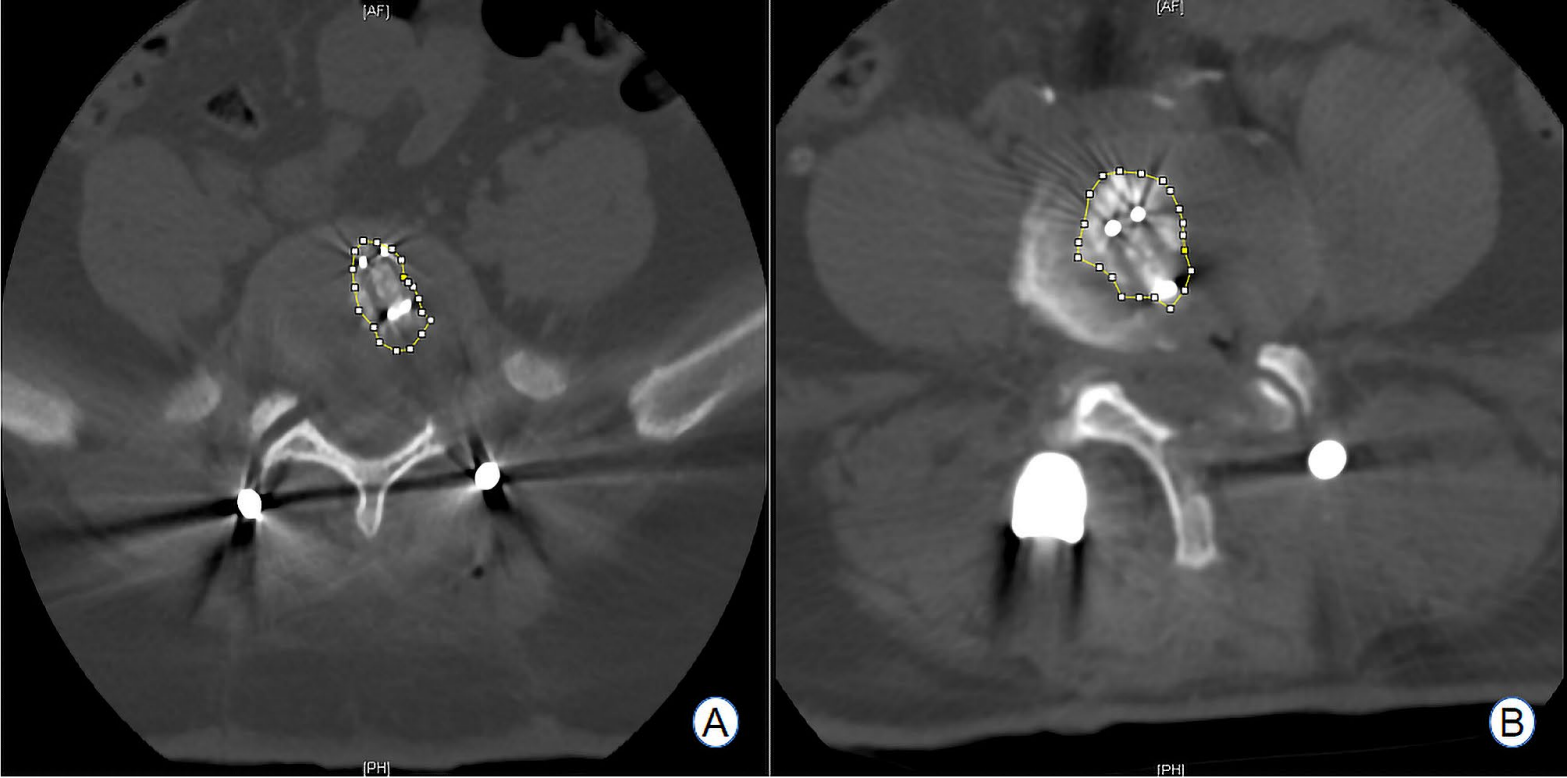
**A** Area of implanted bone measured before and after surgery in a patient of the Endo-TLIF group; **B** Area of implanted bone measured before and after surgery in a patient of the ULIF group

Both patient groups underwent follow-up assessments, which included a review of the modified Brantigan score, intervertebral fusion rate, and postoperative imaging results at 6 and 12 months post-surgery (refer to Table 2; Fig. 15). The differences at each time point were not statistically significant (*P* > 0.05). The changes in the volume of the spinal canal in the same plane as the target lumbar intervertebral space, as determined through MRI examination and measured using Image J software, were statistically significant when comparing postoperative measurements to preoperative values (*P* < 0.05)(see Fig. 16). However, there was no statistically significant difference in the change in the cross-sectional area of the vertebral canal at the same level of the intervertebral space (*P* > 0.05). The assessment of changes in the area of bone grafts, as determined by postoperative CT examination using Image J software (see Fig. 17), revealed that the area of bone grafting in the ULIF group was significantly larger than that in the Endo-TLIF group (*P* < 0.05).

Patients were subjected to evaluations before surgery and at 1 week, 3 months, and 12 months following the surgical intervention. Efficacy at each time point was evaluated by the VAS score and ODI index. Importantly, there were no statistically significant differences in VAS scores and ODI indices between the two patient groups before surgery, indicating the groups’ comparability (see Table 1) (*P* > 0.05). However, when comparing the values to those before surgery, significant differences were observed in the VAS scores and ODI indices at 1 week, 3 months, and 12 months post-surgery (*P* < 0.05) (see Table 2). The VAS and ODI indices of the two groups of patients at each time point were analyzed using analysis of variance. The group effects, interaction effects, and time effects are shown in Fig. 15.

## Discussion

Minimally invasive lumbar fusion techniques have gained increasing popularity among spinal surgeons. In comparison to traditional lumbar fusion methods, minimally invasive spine surgery offers advantages such as preservation of the vertebral body’s normal structure, reduced surgical trauma, shorter operative times, and accelerated postoperative recovery [7, 10, 11]. However,traditional single-channel spinal endoscopic lumbar fusion has inherent limitations, primarily stemming from the use of single-channel endoscopic techniques, which complicates the procedure due to restricted intraoperative visibility. Moreover, the limited range of operation and the reliance on surgical instruments with a narrow scope can lead to restricted intervertebral space treatment and rapid wear and tear of surgical instruments.

In this context, spinal surgery via the UBE technique was first introduced in 1996 and was gradually improved [12]. UBE, as a minimally invasive procedure, combines the merits of open surgery with traditional minimally invasive surgery. Performing UBE surgery with high-definition visualization effectively safeguards the paravertebral muscles while minimizing damage to the paravertebral bones, joints, and ligaments. Recent years have witnessed substantial progress in unilateral techniques, enabling minimally invasive treatment of conditions such as lumbar disc herniations, severe lumbar spinal stenosis, cervical spinal stenosis, compression fractures, and foraminal nerve compression [4, 13, 14]. Therefore, the ULIF technique has become an effective alternative to conventional lumbar fusion. In essence, the UBE technique represents a minimally invasive approach that offers a comparable scope of operation and field of view to traditional open surgery. Its unique advantages and intrinsic value underscore its broad application and promising prospects. Specifically, the merits of the UBE technique in lumbar spine surgery include the ability to achieve complete bilateral nerve decompression through two small surgical incisions: one serves as an entry point for continuous irrigation and endoscopic observation, while the other allows for instrument manipulation during the procedure [15, 16]. This dual-entry approach compensates for the limitations of traditional percutaneous single-access minimally invasive spine surgery instruments and the constrained surgical field of vision.

Earlier research exposed that the VAS score and ODI index for back pain and leg pain of patients in both groups were significantly improved after surgery compared with those before surgery (*P* < 0.05). Furthermore, there were no significant differences in scores between the two groups at each time point. Additionally, our study found no statistically significant difference in intraoperative bleeding between the two groups (*P* > 0.05). These findings suggest that both ULIF and Endo-TLIF procedures can effectively alleviate pain and promote functional recovery, demonstrating similar treatment effects. Therefore, both ULIF and Endo-TLIF surgeries are equally safe and effective. However, it’s worth noting that patients in the ULIF group in our study experienced shorter operative times and longer hospital stays compared to those in the Endo-TLIF group (*P* < 0.05). It’s important to recognize that intraoperative bleeding and operative time may be influenced by factors such as the surgeon’s operative approach, habits, proficiency, and operating conditions. Despite UBE involving more stripping of muscles and more bone removal, there was increased intraoperative bleeding in some patients of Endo-TLIF group. Some reasons may be as follows: (1) UBE surgery provides a clear intraoperative field of vision, a wide range of surgical instruments, flexible hemostasis operations, and timely application of radiofrequency for hemostasis. (2) Endo-TLIF surgery typically takes longer than UBE surgery. (3) Surgeons’ operating habits and the use of hemostatic drugs during surgery can also influence the amount of bleeding during surgery. Meanwhile, there was no statistical difference in fusion rates between the two surgical methods at 6 months and 12 months post-surgery (*P* > 0.05), confirming the equal effectiveness of both methods. Patients in both groups achieved satisfactory intervertebral fusion following surgery. Nevertheless, a small number of patients in both groups experienced non-fusion at the 12-month postoperative mark, necessitating ongoing monitoring and attention to their intervertebral fusion status for further analysis. If necessary, revision surgery was provided. Several factors may influence intervertebral fusion, including intervertebral infection, endplate management, internal fixation stability, nature of bone graft material, bone graft area, and implantation of a hybrid graft [17, 18]. Although the area of bone graft was numerically higher in the ULIF group than in the Endo-TLIF group, there was no significant difference in the intervertebral fusion rate between the two groups, indicating that the area of intervertebral autologous bone grafting is not the sole determinant of fusion rates [18]. Moreover, our analysis of patients’ lumbar spine imaging data revealed a significant increase in the postoperative spinal canal cross-sectional area (*P* < 0.05) compared to the preoperative period in both groups. However, there was no significant difference in the change in spinal canal area before and after surgery in both groups (*P* > 0.05). This, coupled with the analysis of postoperative clinical outcomes, underscores the effectiveness of both surgical procedures in decompressing the target segments. Besides, the results indicated that the ULIF group had a significantly greater bone grafting range, treatment range, and amount of autologous bone acquisition compared to the Endo-TLIF group. This demonstrates that ULIF surgery can be performed with a clear field of vision, a large range of motion for surgical instruments, and a direct view through the operating channel compared to Endo-TLIF.

In summary, when compared to the traditional Endo-TLIF technique, the ULIF technique offers several advantages: (1) Separate operation of the working channel and observation channel, allowing for unrestricted instrument maneuverability. This setup facilitates precise decompression within a clear and magnified surgical field, offering a wide range of intervertebral space treatment and ample autologous bone acquisition [19]. (2) Wide visualization range with unimpeded access to all positions of the spinal canal, resulting in a substantial decompression range and effective decompression. (3) A relatively shallow learning curve, making it easy to learn and promote the ULIF technique. (4) Simplified intraoperative hemostasis in ULIF patients. However, in comparison to ULIF, Endo-TLIF technology also offers several advantages: (1) It is more minimally invasive than ULIF, which involves more stripping of muscles and bone removal. (2) Endo-TLIF is similar to ULIF in terms of long-term clinical outcomes, fusion rates, and incidence of complications. Endo-TLIF prolongs surgical time but shortens hospital stay. (3) Endo-TLIF causes less trauma and eliminates the need for a drainage tube after surgery, resulting in faster wound healing.The meta-analysis study by H-X Zhu et al. involving 823 patients with a single LSS segment, compared UBE technology with traditional single channel endoscopic technology in terms of surgical time, blood loss, incidence of complications, and admission time. The study indicated that UBE has achieved promising initial clinical results and may serve as a minimally invasive alternative surgery for single segment LSS patients [20]. Zhaoyuan Chen et al.‘s meta-analysis included 24 studies, including 999 patients. Research indicates that the incidence of complications in UBE treatment of LSS is relatively low, mainly due to dural tears [21]. Meanwhile, the meta-analysis study conducted by Jiang Liang et al. included 24 studies, including 999 patients. Research has shown that UBE treatment for LSS is not only a feasible and effective method, but also a worthwhile choice for clinical doctors [22].

Therefore, ULIF has demonstrated effectiveness comparable to that of Endo-TLIF in treating degenerative lumbar spine diseases and achieving interbody fusion. With ongoing advancements in minimally invasive treatment equipment and technology, it is expected that more studies will be conducted on both approaches, leading to further progress. ULIF technology possesses advantages such as a large field of vision, a larger bone grafting range, and favorable clinical outcomes, and combines some advantages of single-channel endoscopy and open surgery. Moreover, In the realm of treating degenerative lumbar spine diseases, Endo-TLIF surgery can be considered as an effective substitute procedure. This operation can be performed in a safe, minimally invasive manner with good clinical results. However, it’s important to note that this study is retrospective and has a limited number of statistical time points. Long-term follow-up and further research are necessary to investigate intervertebral fusion between the two techniques. Additionally, the sample size in this study is limited, with only a portion of eligible patients included. The next steps should involve expanding the sample size, extending the follow-up duration, conducting multi-center collaborative follow-ups, and designing prospective studies to further validate the aforementioned conclusions.

## Conclusion

In summary, the ULIF technique, as a novel spinal endoscopy approach, is a safer and more effective minimally invasive surgical method for treating patients with lumbar spinal stenosis and intervertebral disc herniation. Both surgical methods have their own advantages and disadvantages. With the development of technology and related instruments, the limitations of both techniques can be mitigated to some extent. This progress opens the door for broader applications by a great number of doctors in more fields in the future.

## Data Availability

The datasets used and analysed during the current study available from the corresponding author on reasonable request.
